# An *EAV-HP* Insertion in 5′ Flanking Region of *SLCO1B3* Causes Blue Eggshell in the Chicken

**DOI:** 10.1371/journal.pgen.1003183

**Published:** 2013-01-24

**Authors:** Zhepeng Wang, Lujiang Qu, Junfeng Yao, Xiaolin Yang, Guangqi Li, Yuanyuan Zhang, Junying Li, Xiaotong Wang, Jirong Bai, Guiyun Xu, Xuemei Deng, Ning Yang, Changxin Wu

**Affiliations:** National Engineering Laboratory for Animal Breeding and Key Laboratory of Animal Genetics, Breeding, and Reproduction of the Ministry of Agriculture, China Agricultural University, Beijing, China; Stanford University School of Medicine, United States of America

## Abstract

The genetic determination of eggshell coloration has not been determined in birds. Here we report that the blue eggshell is caused by an *EAV-HP* insertion that promotes the expression of *SLCO1B3* gene in the uterus (shell gland) of the oviduct in chicken. In this study, the genetic map location of the blue eggshell gene was refined by linkage analysis in an *F_2_* chicken population, and four candidate genes within the refined interval were subsequently tested for their expression levels in the shell gland of the uterus from blue-shelled and non-blue-shelled hens. *SLCO1B3* gene was found to be the only one expressed in the uterus of blue-shelled hens but not in that of non-blue-shelled hens. Results from a pyrosequencing analysis showed that only the allele of *SLCO1B3* from blue-shelled chickens was expressed in the uterus of heterozygous hens (O*LC/O*N). *SLCO1B3* gene belongs to the organic anion transporting polypeptide (OATP) family; and the OATPs, functioning as membrane transporters, have been reported for the transportation of amphipathic organic compounds, including bile salt in mammals. We subsequently resequenced the whole genomic region of *SLCO1B3* and discovered an *EAV-HP* insertion in the 5′ flanking region of *SLCO1B3*. The *EAV-HP* insertion was found closely associated with blue eggshell phenotype following complete Mendelian segregation. *In situ* hybridization also demonstrated that the blue eggshell is associated with ectopic expression of *SLCO1B3* in shell glands of uterus. Our finding strongly suggests that the *EAV-HP* insertion is the causative mutation for the blue eggshell phenotype. The insertion was also found in another Chinese blue-shelled breed and an American blue-shelled breed. In addition, we found that the insertion site in the blue-shelled chickens from Araucana is different from that in Chinese breeds, which implied independent integration events in the blue-shelled chickens from the two continents, providing a parallel evolutionary example at the molecular level.

## Author Summary

The eggshell color of birds is of wide interest, but the molecular basis remained unknown until our discovery, reported here. The blue eggshell is found not only in wild birds but also in domestic fowls. In this study, we identified that blue eggshell in chickens from different geographical regions is caused by a ∼4.2 kb *EAV-HP* insertion in the 5′ flanking region of *SLCO1B3*. The *EAV-HP* insertion in chicken is a derived mutation in domestic chickens. The genetic determination of blue eggshell in other birds requires further investigation. We also found that the *EAV-HP* insertions in the chickens from China and America were separate integration events, which presents us with a parallel molecular evolution example driven by artificial selection.

## Introduction

Avian eggshell coloration is the result of crypsis or mimetism and plays important roles in filtering solar radiation and strengthening the eggshell [1]. Blue eggshell color has been proposed as post-mating signals of female phenotypic quality to their mates and is related to fitness of the offspring due to the antioxidant of biliverdin, a predominant pigment for blue eggs [2], [3]. Blue eggshells can be found not only in some wild birds, e.g. eastern bluebird [4], blue-footed booby [5], and pied flycatcher [6], but also in domestic birds such as Japanese quail [7], chickens [8] and ducks [9].

Brown and white are the two major eggshell colors in chickens. Protoporphyrin-IX, biliverdin, and biliverdin zinc chelate are the main pigments of the eggshell [10] and several blue egg laying breeds have been reported worldwide [11], [12]. The Araucana, an indigenous breed from Chile, was the first chicken breed described to lay blue eggs [8], and has been frequently used in genetic studies of the blue eggshell phenotype. In China, Dongxiang and Lushi chickens are representative breeds laying blue eggs and show dominant inheritance as that in Araucana. However, the blue eggshell phenotype has not been fixed in these three breeds which still produce brown eggs at low frequency.

**Figure 1:**
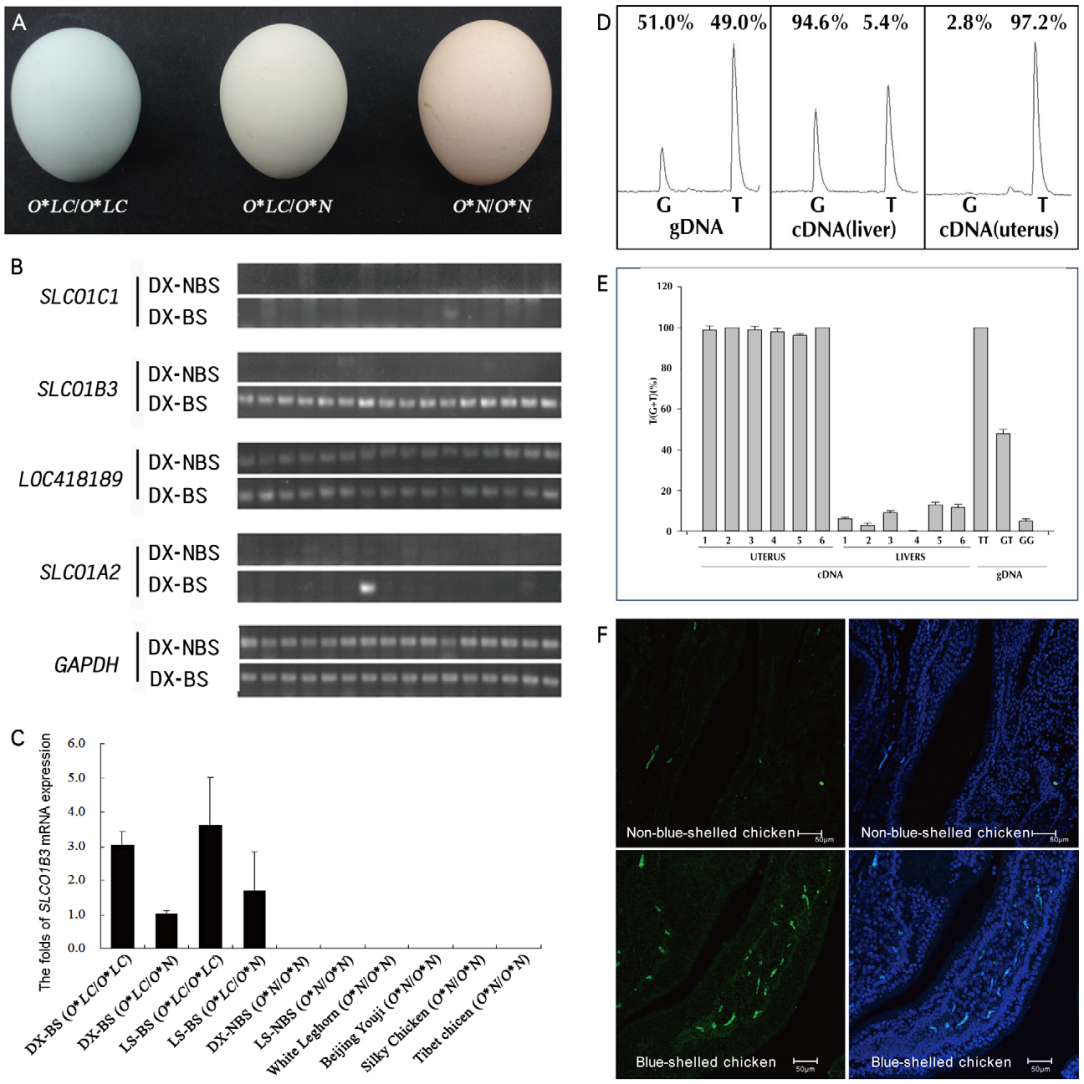
The eggshell color and the expression of *SLCO1B3* in the uterus of blue-shelled and non-blue-shelled chickens. (A) Eggshell colors of homozygous blue-shelled (O*LC/O*LC), heterozygous blue-shelled (O*LC/O*N) and brown-shelled (O*N/O*N) of Dongxiang chickens. (B) The expression analysis of the four genes which are located in the refined region between markers L4 and L5 in the uterus of Dongxiang blue-shelled hens (n = 16) and Dongxiang non-blue-shelled hens (n = 16) by RT-PCRs. DX-BS: blue-shelled Dongxiang, DX-NBS: non-blue-shelled Dongxiang. (C) Analysis of *SLCO1B3* expression in the uterus. Expression data were presented as fold relative to heterozygous blue-shelled Dongxiang chickens (O*LC/O*N) by the comparative C_t_ method (2^−ΔΔCt^). *SLCO1B3* is exclusively expressed in blue-shelled chickens, and the amount of *SLCO1B3* transcripts in homozygous blue-shelled chicken (O*LC/O*LC) is approximately two to three folds of that in heterozygous individuals (O*LC/O*N). DX-BS: blue-shelled Dongxiang, LS-BS: blue-shelled Lushi, DX-NBS: non-blue-shelled Dongxiang, LS-NBS: non-blue-shelled Lushi. (D) Micrographs of cDNA *in situ* hybridization for *SLCO1B3* mRNA in the uterus from blue-shelled and non-blue-shelled chickens. (E) Differential expression of *SLCO1B3* transcript in the uterus from blue-shelled heterozygotes using genomic DNA (gDNA) as control. The polymorphic position *g. 67334934* G>T was used to monitor differential expression using pyrosequencing. T and G at this position correspond to blue-shell and non-blue-shell alleles, respectively. Due to two Ts next to the SNP at 3′end, the peaks of T in the schema contain three Ts including one T from blue-shell allele and two Ts from non-blue-shell allele. The percent expression on the peaks for T and G are the T or G at *g. 67334934* G>T. (F) Summary of the detection of differential expression in uterus and liver from six heterozygotous blue-shelled (O*LC/O*N) birds.

Blue eggshell color exhibits an autosomal dominant inheritance and eggs laid by homozygotes are a darker blue than those from heterozygotes (Figure 1A). In 1933, Punnett firstly reported that blue or green shell appearance of the Araucana was determined by a single genetic factor, traditionally denoted as oocyan (*O*) [8]. A series of linkage analysis involving *O* have been performed with *O* affirmatively mapped to the short arm of chromosome 1 [12]–[16], and closely linked to *ev1* and *P* which was identified as *SRY* (*sex determining region Y)-box 5* (*SOX5*) [12], [13], [16], [17]. In the region around *ev1*, two single nucleotide polymorphisms (SNPs) (rs15297163 and rs15297165) were found to be highly associated with the blue eggshell phenotype [18]. A 1.8 Mb genomic interval harboring the *O* gene was defined in an *F_2_* resource population [19]. The localization of the *O* was further refined to the vicinity of ss244244378 by linkage and association analysis [20]. The ss244244378 is very close to the two SNPs reported by Zhao et al. [18] with a physical distance of 0.12 Mb implies that the region around the three SNPs is mostly like to harbor the blue eggshell gene. Combined mapping information from traditional breeds and Chilean village chickens allowed the *O* to be fine mapped to two small regions (Gga 1:67.25–67.28 Mb, Gga 1:67.28–67.32 Mb) [21].

In the present study we found that the blue eggshell phenotype in chickens is caused by a retrovirus insertion in the 5′ flanking region of *SLCO1B3* coding a membrane transporter OATP1B3 which is responsible for transporting amphipathic organic compounds including bile salt.

## Results

### Linkage analysis of Chicken blue eggshell gene

**Figure 2:**
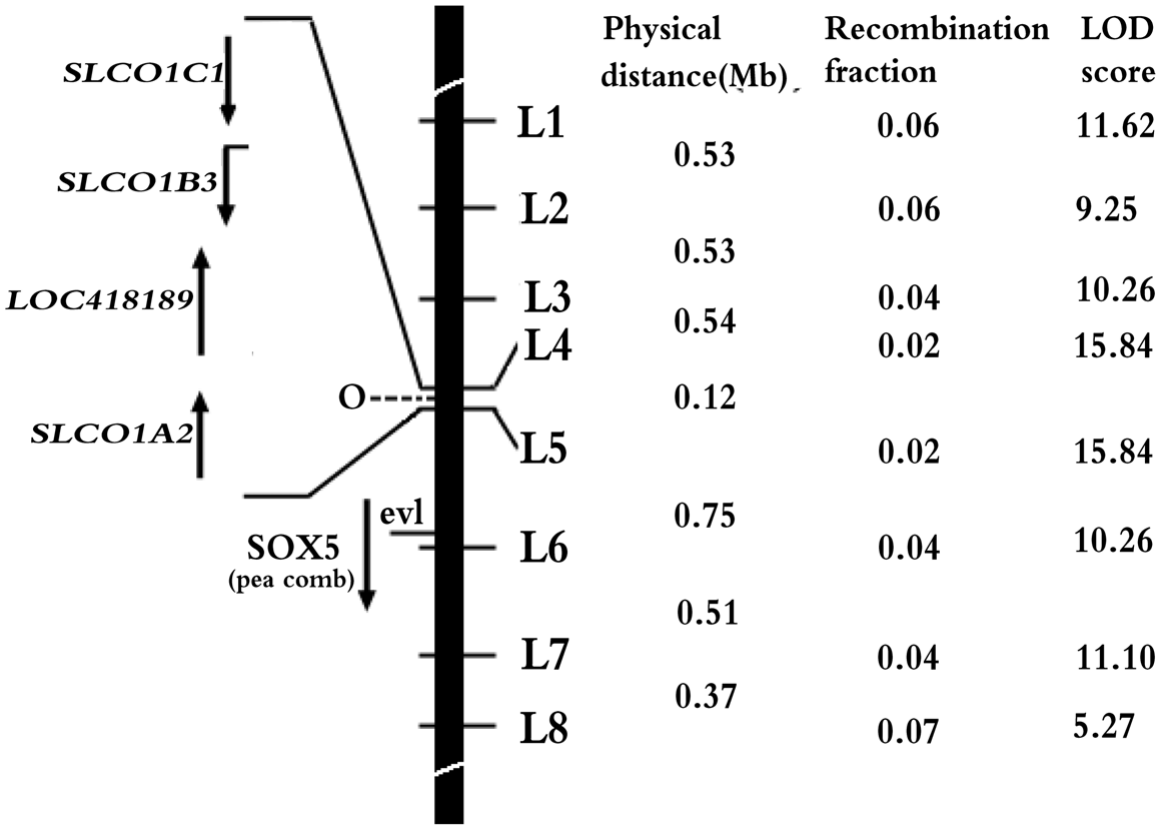
Refined localization of *O* on chicken chromosome 1. The black bar represents the short arm of chicken chromosome 1. Numbers in middle column are recombination fractions between markers and *O*, the corresponding LOD scores are shown in the last column. Here, *O* is assigned to the interval of L4 and L5 at Chr1: 67296991–67419904. Four candidate genes in the interval, *EAV-HP* insertion with recombination fraction and LOD in the parentheses, *SRY (sex determining region Y)-box 5* (*SOX5*) for pea comb phenotype [17], *ev1* marker [16] are indicated at the left of the black bar.

A linkage analysis was performed in an *F_2_* resource population segregating for the *O* gene to refine the location of chicken blue shell gene in the present study. Eight molecular markers in the candidate region were used for linkage analysis (Table S1). By two-point analysis, the *O* gene was mapped in the region between marker L4 and L5 which were the closest flanking markers to *O* with recombination rate being both 0.02 (LOD = 15.84) (Figure 2). Fifteen SNP markers between L4 and L5 were further genotyped in the *F_2_* resource population to narrow the mapping region and the *O* was finally located in a ∼120 kb region from 67296991 bp to 67416784 bp on chromosome 1 on the UCSC chicken genome (May 2006 assembly) (Table S1) and no recombination was found between the blue eggshell phenotype and the markers within the region.

### Specific expression of *SLCO1B3* in uterus of blue-shelled chicken

Totally, four genes (*SLCO1C1*, *SLCO1B3*, *LOC418189* and *SLCO1A2*) were found in the ∼120 kb interval by a MapView search (http://www.ncbi.nlm.nih.gov/mapview/) (Table S2). The uterus is where pigment is secreted to eggshell. We performed expression analysis for the four candidate genes in the uterus of blue-shelled (n = 16) and brown-shelled (n = 16) Dongxiang hens by RT-PCR. We found that *SLCO1B3* was the only gene expressed specifically in the uterus of blue-shelled Dongxiang chickens (Figure 1B), thus we measured its expression in the uterus in 8 blue-shelled (4 O*LC/O*LC and 4 O*LC/O*N. O*LC is for the blue-shell allele in the two Chinese breeds, Dongxiang and Lushi Chicken and O*N denotes the non-blue-shell allele or wild-type allele) and 4 brown-shelled (O*N/O*N) Dongxiang chickens, 6 blue-shelled (3 O*LC/O*LC and 3 O*LC/O*N) and 6 brown-shelled (O*N/O*N) Lushi chickens, and 24 chickens from three brown-shelled and one white-shelled breeds (O*N/O*N, 6 chickens per breed) by real-time PCR. All blue-shelled chickens expressed the gene in the uterus while non-blue-shelled chickens did not (Figure 1C). In addition, expression of *SCLO1B3* was 2 to 3 fold higher in homozygous blue-shelled chickens than in heterozygous blue-shelled Dongxiang and Lushi individuals (Figure 1C). Fluoscence labeled cDNA *in situ* hybridization demonstrated that the transcripts of *SLCO1B3* were only expressed in the uterus of blue-shelled but not brown-shelled hens (Figure 1D). These results suggest that *SLCO1B3* is the causative gene for blue eggshell in the chicken.

### Allele-specific expression of *SLCO1B3*

We found a SNP (*g.67334934* G>T) in exon 5 of *SLCO1B3* gene by sequencing the coding region and the SNP presented complete association with the blue eggshell phenotype in the Dongxiang chicken by genotyping it in Dongxiang blue-shelled and brown-shelled chickens. With six heterozygous individuals produced by mating a homozygous Dongxiang blue-shelled male with a White Leghorn female, the allelic expression of *SLCO1B3* gene was demonstrated by RT-PCR analysis and pyrosequencing. More than 95% of the transcripts expressed in the uterus originated from the T allele corresponding to the blue-shell allele (Figure 1E). This means the expression of the gene is regulated by a *cis*-acting element. Surprisingly, its expression in liver is also allele specific, and ∼95% of the transcripts in liver come from the G allele which is non-blue-shell allele (Figure 1F).

### An *EAV-HP* insertion is completely associated with blue eggshell phenotype

We sequenced the genomic region of *SLCO1B3* in order to reveal the potential causative mutation of the gene with 5 blue-shelled and 5 brown-shelled Dongxiang chickens. Twenty-one SNPs evenly covering the whole genomic region (∼24 kb) of *SLCO1B3* were taken for genotyping in 353 chickens from 3 blue-shelled breeds (Araucana, Dongxiang and Lushi) and 9 non-blue-shelled breeds. However, none of the SNPs was found to be in complete linkage disequilibrium with blue eggshell (Table 1).

**Table 1:** Distribution of allelic frequencies of SNPs and *EAV-HP* in *SLCO1B3* in several blue-shelled and non-blue-shelled breeds.

	Description	Blue-shelled^a^			Brown-shelled								White-shelled
		Dongxiang	Lushi	Araucana	Dongxiang	Lushi	Red Jungle Fowl	Rhode Island Red	Dwarf	Luxi Game	Silkie	Tibetan	White Leghorn
N		33	29	7	43	30	31	30	30	30	30	30	30
1B3_1	*g.67320217A>G*	0.92 (A)	0.85 (A)	0.07 (A)	0.00 (A)	0.30 (A)	0.21 (A)	0.03 (A)	0.46 (A)	0.56 (A)	0.08 (A)	0.27 (A)	0.00 (A)
1B3_2	*g.67320779A>G*	0.92 (A)	0.85 (A)	0.07 (A)	0.00 (A)	0.29 (A)	0.37 (A)	0.03 (A)	0.47 (A)	0.60 (A)	0.17 (A)	0.34 (A)	0.00 (A)
1B3_3	*g.67322980C>T*	0.92 (C)	0.83 (C)	0.01 (C)	0.00 (C)	0.18 (C)	0.07 (C)	0.23 (C)	0.08 (C)	0.05 (C)	0.00 (C)	0.18 (C)	0.48 (C)
***EAV-HP*** b	***67324641–67324642***	**0.92 (+)** c	**0.83 (+)** c	**0.79 (+)** c	**0.00 (+)**	**0.00 (+)**	**0.00 (+)**	**0.00 (+)**	**0.00 (+)**	**0.00 (+)**	**0.00 (+)**	**0.00 (+)**	**0.00 (+)**
1B3_4	*g.67325964G>C*	0.97 (G)	0.85 (G)	0.79 (G)	0.63 (G)	0.65 (G)	0.66 (G)	0.75 (G)	0.45 (G)	0.43 (G)	0.87 (G)	0.70 (G)	0.52(G)
1B3_5	*g.67328567A>G*	0.97 (G)	0.83 (G)	0.00 (G)	0.00 (G)	0.17 (G)	0.13 (G)	0.00 (G)	0.03 (G)	0.15 (G)	0.48 (G)	0.09 (G)	0.17 (G)
1B3_6	*g.67330251C>T*	1.00 (C)	1.00 (C)	0.00(C)	1.00 (C)	0.38 (C)	0.48 (C)	0.18 (C)	0.03 (C)	0.28 (C)	0.33 (C)	0.43 (C)	0.00 (C)
1B3_7	*g.67330635C>T*	0.92 (T)	0.83 (T)	0.00(T)	0.00 (T)	0.02 (T)	0.05 (T)	0.00 (T)	0.00 (T)	0.00 (T)	0.00 (T)	0.00 (T)	0.00 (T)
1B3_8	*g.67332494A>G*	1.00 (A)	0.97 (A)	1.00 (A)	1.00 (A)	0.43 (A)	0.31 (A)	0.97 (A)	0.50 (A)	0.15 (A)	0.38 (A)	0.82 (A)	0.45 (A)
1B3_9	*g.67333488C>T*	0.92 (T)	0.83 (T)	0.00 (T)	0.00 (T)	0.03 (T)	0.13 (T)	0.00 (T)	0.00 (T)	0.00 (T)	0.00 (T)	0.00 (T)	0.00 (T)
1B3_10	*g.67334934G>T*	0.92 (T)	0.83 (T)	0.00(T)	0.00 (T)	0.07 (T)	0.08 (T)	0.00 (T)	0.00 (T)	0.23 (T)	0.13 (T)	0.02 (T)	0.00 (T)
1B3_11	*g.67335932T>C*	0.97 (T)	0.85 (T)	0.14 (T)	0.63 (T)	0.53 (T)	0.61 (T)	0.78 (T)	0.43 (T)	0.32 (T)	0.63 (T)	0.54 (T)	0.10 (T)
1B3_12	*g.67336084C>T*	0.92 (T)	0.83 (T)	0.00(T)	0.00 (T)	0.03(T)	0.07 (T)	0.00 (T)	0.00 (T)	0.00 (T)	0.13 (T)	0.02 (T)	0.00 (T)
1B3_13	*g.67336453G>T*	0.92 (T)	0.83 (T)	0.00 (T)	0.00 (T)	0.03 (T)	0.22 (T)	0.00 (T)	0.00 (T)	0.00 (T)	0.13 (T)	0.02 (T)	0.00 (T)
1B3_14	*g.67336599C>T*	0.92 (T)	0.83 (T)	0.00(T)	0.00 (T)	0.03 (T)	0.21 (T)	0.00 (T)	0.00 (T)	0.02 (T)	0.13(T)	0.02 (T)	0.00 (T)
1B3_15	*g.67336867A>T*	0.92 (T)	0.83 (T)	0.00(T)	0.00 (T)	0.06 (T)	0.35 (T)	0.00 (T)	0.00 (T)	0.00 (T)	0.13 (T)	0.02 (T)	0.00 (T)
1B3_16	*g.67337145A>G*	0.92 (G)	0.83 (G)	0.00(G)	0.00 (G)	0.03 (G)	0.22 (G)	1.00 (G)	0.00 (G)	0.00 (G)	0.03 (G)	0.00 (G)	0.55 (G)
1B3_17	*g.67338442C>G*	0.92 (G)	0.83 (G)	0.00(G)	0.00 (G)	0.03 (G)	0.23 (G)	0.00 (G)	0.00 (G)	0.00 (G)	0.13 (G)	0.00 (G)	0.55 (G)
1B3_18	*g.67339003C>T*	0.96 (T)	0.97 (T)	0.00(T)	0.22 (T)	0.23 (T)	0.24 (T)	0.00(T)	0.03 (T)	0.18 (T)	0.30 (T)	0.05 (T)	0.55(T)
1B3_19	*g.67339848A>G*	0.92 (G)	0.85 (G)	0.00 (G)	0.00 (G)	0.13 (G)	0.32 (G)	0.00 (G)	0.00 (G)	0.02 (G)	0.10 (G)	0.00 (G)	0.00 (G)
1B3_20	*g.67340370C>T*	0.92 (T)	0.83 (T)	0.00 (T)	0.00 (T)	0.02 (T)	0.26 (T)	0.17 (T)	0.00 (T)	0.00 (T)	0.10 (T)	0.00 (T)	0.00 (T)
1B3_21	*g.67342640A>G*	0.92 (A)	0.85 (A)	0.00 (A)	0.00 (A)	0.10 (A)	0.00 (A)	0.00(A)	0.00 (A)	0.02 (A)	0.27 (A)	0.02 (A)	0.52 (A)

a Both homozygous and heterozygous hens in these populations lay blue-shelled eggs. Dongxiang and Lushi blue-shelled chickens are from China and Araucana chickens are from South America.

b *EAV-HP* is the only one mutation showing complete association with blue eggshell phenotype. The “+” sign indicates that *EAV-HP* insertion is positive, while the “−” sign indicates no *EAV-HP*.

c All detected blue-shelled chickens carry EAV-HP insertion and the frequency of “+” allele is not equal to 1 in blue-shelled groups because some birds are heterozygotes (+/−).

**Figure 3:**
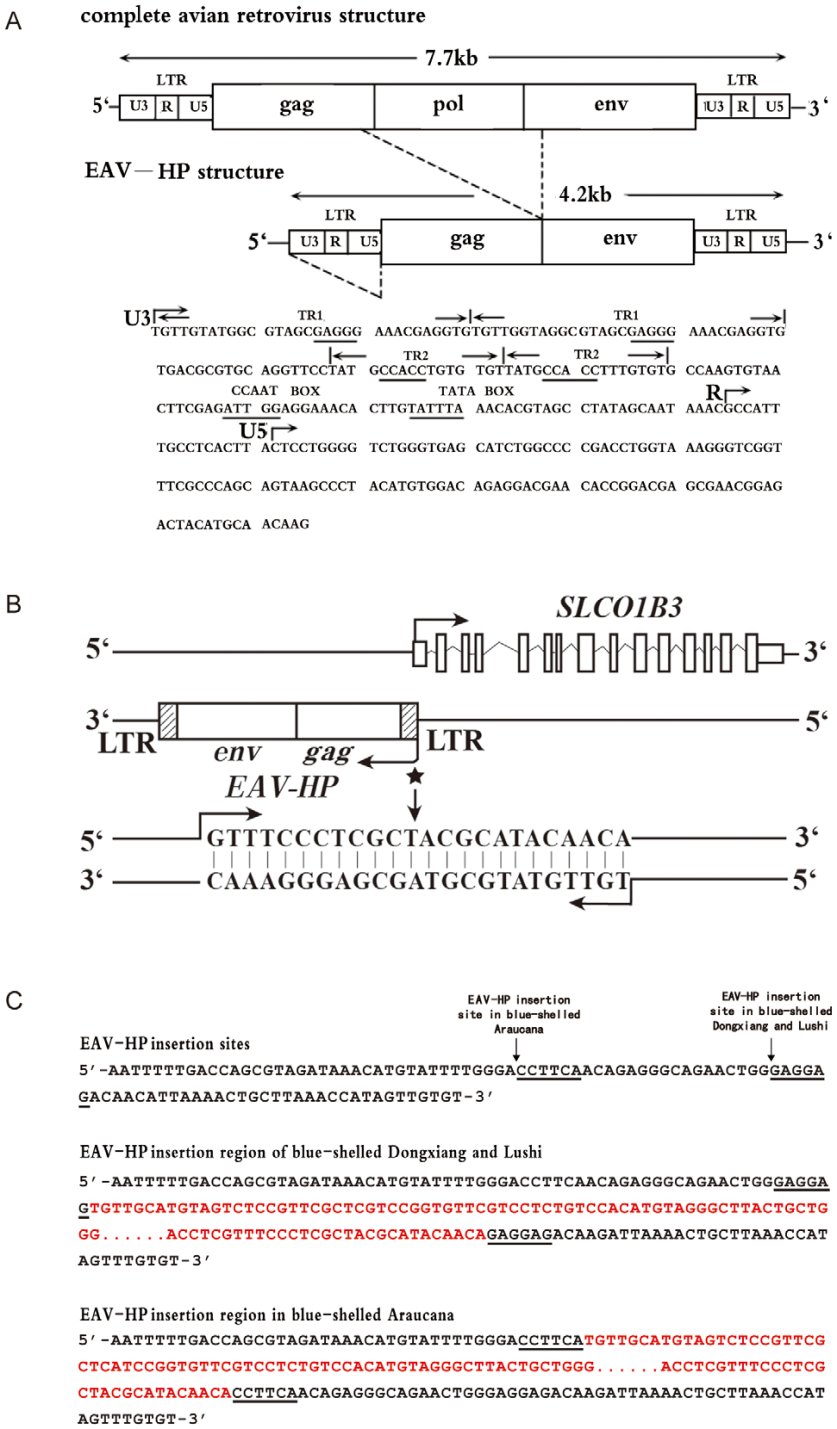
The *EAV-HP* insertion in blue-shelled chickens. (A) Schematic diagram of the relationship of a complete avian leukosis virus and the *EAV-HP* retrovirus. Some putative *cis*-regulatory elements in long terminal repeat (LTR) are underlined. The diagram was redrawn by referencing Sacco et al. [22]. (B) Arrangement of *EAV-HP* retrovirus and *SLCO1B3* in chicken genome. *EAV-HP* integrates into the 5′ end of *SLCO1B3* in inverted orientation. When *SLCO1B3* is transcribed, an extra 24-bp sequence from the *EAV-HP* is also compiled into *SLCO1B3* transcript. The asterisk indicates the site of 24-bp sequence from *EAV-HP*, and arrows indicate the direction of transcription. (C) *EAV-HP* junction sites in blue-shelled chickens of Dongxiang, Lushi and Araucana breeds. The arrows show the *EAV-HP* inserted sites and the underlined sequences are the *EAV-HP* integration specific sequences. The sequences in red denote the inserted *EAV-HP*.

We subsequently cloned the 5′UTR (GenBank accession number: JN381032) of *SLCO1B3* by 5′ RACE in a blue-shelled (O*LC/O*LC) and a brown-shelled (O*N/O*N) Dongxiang chicken and an extra 24 bps were found at the beginning of 5′UTR end in blue-shelled Dongxiang chicken (Figure S1). We further sequenced 5 kb upstream of the promoter using 5 blue-shelled (O*LC/O*LC) and 5 brown-shelled (O*N/O*N) Dongxiang chickens. A ∼4.2 kb insertion adjacent to 5′UTR containing the extra 24 bps was found in the blue-shelled but not in the brown-shelled chickens. The sequence of the ∼4.2 kb insertion (GenBank accession number: JF837512) represents an incomplete retrovirus and shows 95.8% identity with the sequence of the avian *EAV-HP* retrovirus (EMBL accession number: AJ238124) [22]. A typical proviral structure consists of *gag*, *pol* and *env* flanked by long terminal repeat (LTR), which are arranged in the order of 5′LTR-*gag-pol-env*-LTR3′ [22]. Here, the inserted retrovirus is absent of the whole *pol* gene and part of *gag* and *env* (Figure 3A). The retrovirus was integrated into the blue-shelled chicken genome in an inverted orientation (Figure 3B) at Chr1: 67324641–67324642. We also found that the *EAV* encompassed some promoter elements by sequence analysis, indicating its expression promotion activities (Figure 3A).

A wide-range survey of the *EAV-HP* insertion was performed in 705 chickens from 12 worldwide breeds and the *F_2_* resource population (Table 2) using diagnostic PCR test. The results that the *EAV-HP* insertion is completely associated with the blue eggshell phenotype provide strong evidence that the mutation is causative.

**Table 2:** *EAV-HP* insertion distributions in various populations.

Breed or population		n	*EAV-HP* insertion^a^		
			+/+	+/−	−/−
**Blue-shelled**					
*Dongxiang* (O*LC/-)		*111*	*100*	*11*	*0*
*Resource family*	*F_0_* (O*LC/O*LC)	*5*	*5*	*0*	*0*
	*F_1_* (O*LC/O*N)	*56*	*0*	*56*	*0*
	*F_2_* (O*LC/-)	*121*	*39*	*82*	*0*
Lushi (O*LC/-)		30	21	9	0
Araucana (O*LA b/-)		8	5	3	0
**Brown-shelled**					
Red jungle fowl (O*N/O*N)		31	0	0	31
Dongxiang (O*N/O*N)		28	0	0	28
Resource family	*F_0_* (O*N/O*N)	20	0	0	20
	*F_2_* (O*N/O*N)	25	0	0	25
Lushi (O*N/O*N)		30	0	0	30
Beijing You (O*N/O*N)		30	0	0	30
Silkies (O*N/O*N)		30	0	0	30
Tibetan (O*N/O*N)		30	0	0	30
Luxi Game (O*N/O*N)		30	0	0	30
Gushi (O*N/O*N)		30	0	0	30
Dwarf (O*N/O*N)		30	0	0	30
Rhode Island Red (O*N/O*N)		30	0	0	30
**White-shelled**					
White Leghorn (O*N/O*N)		30	0	0	30

a The “+” sign indicates a positive result of insertion, and the “−” denotes no insertion.

b The “O*LA” is for the blue-shell allele in Araucana.

### Independent *EAV-HP* insertion events in blue-shelled chickens from China and Chile

In order to elucidate whether the blue-shelled chickens from China and Chile have the same origin for the genotypic mutation, we further sequenced the *EAV-HP* insertion regions in a homozygous Araucana and a homozygous blue-shelled Lushi chicken. The *EAV-HP* insertion was found in both samples and the alignments of Araucana and Lushi to Dongxiang blue-shelled chicken showed the identity of the inserted *EAV-HP* being around 97%. Interestingly, the insertion sites in Araucana are different from that in the two Chinese blue-shelled chickens. The break point for *EAV-HP* insertion in blue-shelled Araucana is located at 23 bp upstream to that in the two Chinese breeds (Figure 3C). We sequenced the junction sites in homozygous blue-shelled chickens of Araucana (n = 5), Lushi (n = 5) and Dongxiang (n = 5) and confirmed the insertion sites in Dongxiang and Lushi are the same but different from that in Araucana.

We also typed 21 SNPs in genomic region of *SLCO1B3* in multiple breeds of blue-shelled and non-blue-shelled chicken breeds. It is obvious to see that the *EAV-HP* insertions in blue-shelled chickens from the two continents were embedded in two distinguished different haplotypes (Table S3), which supports independent integrations accounting for the blue shelled phenotypes.

## Discussion

In birds, eggshell color is a variable Mendelian trait. Colored eggshell could function as avoiding predation through either crypsis or aposematism, distinguishing from brood parasitism, reinforcing eggshell strength, regulating egg temperature, combating harmful solar radiation and sending sexually selected signal to males [2], [23]. However, molecular mechanism of all kinds of eggshell color formation is poorly understood to date. Here, we demonstrate that a ∼4.2 kb *EAV-HP* insertion at upstream of *SLCO1B3* is responsible for blue eggshell phenotype in the chicken.

By linkage analysis, we fine mapped the *O* locus to a 120 kb region, where four candidate genes of *SLCO1C1*, *SLCO1B3*, *LOC418189* and *SLCO1A2* are located. These genes are all members of organic anion transporting polypeptides (OATP, gene symbol *SLCO*). Functionally, the OATPs serve as membrane transporters that mediate a wide range of sodium-independent transport of amphipathic organic compounds, such as some endobiotic compounds of bile salts, eicosanoids, sterioids, thyroid hormones and some xenobiotic compounds of anionic oligopeptides, organic dyes, toxins, and drugs [24]. *SLCO1B3* codes a membrane transporter OATP1B3 which is considered a liver-specific transporter and is highly expressed in liver where it transports a wide range of substrates including bile salts [24], [25]. A genome-wide association study (GWAS) for serum bilirubin levels also showed *SLCO1B3* is a plausible candidate gene responsible for changes in bilirubin levels in humans [26]. As blue egg is colored mainly by deposition of biliverdin on the eggshell and biliverdin is just one component of the bile salts, the expression of the *SLCO1B3* in uterus could enhance transportation of biliverdin to eggshell. In this study, we found that *SLCO1B3* is exclusively expressed in shell gland of uterus of blue-shelled chickens rather than in that of brown- or white-shelled chickens, which supports that the gene plays a pivotal role for coloration of blue eggs.

Regulatory mutations demonstrate an important role for phenotypic diversity which may be explained by *cis*-acting elements [17], [27]–[34]. The effect of endogenous retrovirus (*ERV*) on hosts is extensive. It can unfavorably influence certain production traits, i.e. egg production, egg weight and body weight [35], induce lymphoid or erythroid leukosis and a variety of tumors [35], and cause some phenotype variants, i.e. dilute coat color mutation [36] and hairless mutation in mice [37], recessive white [38], henny-feathering mutation [39], and the sex-linked late-feathering mutation [40] in chickens and outheld wing mutation in *Drosophila melanogaster* [41]. *ERV* could alter splicing patterns of transcript to produce variants such as the recessive white mutation in the chickens [38]. ERV could also promote expression of genes in alternative tissues, which is associated with activity of LTR which contains promoter and/or enhancer sequences responsible for transcription of virus genes [22] and may induce expression of flanking genes. Avian lymphoid leukosis and the henny-feathering mutation are respectively related to activation of *c-myc* in B cell and aromatase in the extrogonadal tissues by LTR [35], [39]. We found an *EAV-HP* inserted in 5′ flanking region of *SLCO1B3* in reverse orientation. The LTR of *EAV-HP* could induce expression of the downstream gene (*SLCO1B3*) by its bidirectional promoter activity [22]. Moreover, 5′UTR of *SLCO1B3* transcripts from the blue-shell allele containing the 24 bp *EAV-HP* partial sequence implies that the expression of *SLCO1B3* in blue-shelled chickens is closely related to the insertion.

Blue eggshells are also seen in other avian species, such as domestic duck, Japanese quail and wild birds [4]–[7], [9]. The genetic pattern of duck blue egg is similar to that of chicken, displaying a dominant phenotype determined by a single gene [42]. However, *SLCO1B3* is not expressed in the uterus of blue-shelled and non-blue-shelled ducks, and the *EAV-HP* insertion was not found in the homologous region in duck (Figure S2, Primers in Table S4). Thus, the causative gene for blue eggshell in ducks may be different from that in chickens. Moreover, the genetic pattern in the chicken is also different from that for Japanese blue-eggshell quail which arise from a recessive mutation *ce* [7]. Because there is no record showing that the two ancestral species of domestic chickens, red jungle fowl and grey jungle fowl [33], lay blue eggs, we may conclude that the causative *EAV-HP* insertion for blue eggshell is a derived mutation in the domestic chicken.

China and Chile are two countries reported for having indigenous blue-shelled chicken breeds. Araucana from Chile, Dongxiang and Lushi from China got the blue eggshell phenotype and were all bred for several hundred years. Analysis with mtDNA showed both Indo-European and Asian origins of Chilean and Pacific chickens and blue/green-shell trait in the Araucana did not originated from ancient pacific/pre-Columbian chickens [43]. It is noted in the present study that though all these blue-shelled chickens had the *EAV-HP* insertion, the *EAV-HPs* inserted into two different genomic sites in the 5′ flanking region of *SLCO1B3* in the blue-shelled chickens from the two countries (Figure 3C) and the *EAV-HP* insertion in blue-shelled Araucana embedded in a haplotype which is distinctly different from the corresponding haplotype from blue-shelled Lushi and Dongxiang chickens (Table S3). Here, we provide unambiguous evidences that the genetic basis of blue shell phenotype in Araucana is different from that in Chinese blue-shelled breeds, indicating independent originations of the trait in different continents. Due to the blue eggshell mutation having been artificially selected for consumption and variable eggshell color types for human requirements, the separated insertion events present us another parallel evolution case at the molecular level under adaptive selection by humans.

## Materials and Methods

### Animals

Two Chinese indigenous blue-shelled chicken breeds, Dongxiang and Lushi, and an American blue-shelled breed, Araucana, were used in the present study. Dongxiang chicken is from Dongxiang town, Jiangxi province of China. It is characterized by blue eggshell, single comb and black feather. Historically Dongxiang chicken is selected for blue eggshell, however, the trait has not been fixed to date. Lushi chicken is another local breed laying blue-shelled egg from Lushi town, Henan province of China. Because Lushi chicken has not been systematically bred, some appearance traits, eggshell color, as well as feather color does not show homogeneity. Araucana is an indigenous breed from Chile of South America. Besides blue-shelled egg, two distinguishing characteristics of Araucana breed are rumpless and tufts of feathers which protrude from each side of neck.

In the present study, Dongxiang chicken and Lushi chicken were collected from Jiangxi Hualv breed poultry conservation farm and Henan Sanmenxia Lushi chicken farm, respectively. The blood samples of Araucana were obtained from members of the Araucana Club of America. We also collected 9 non-blue-shelled chicken breeds including Red Jungle Fowl, White Leghorn, Rhode Island Red, Beijing You, Silkie, Tibetan, Luxi Game, Gushi and Dwarf (a commercial layer line in China).

A three-generation *F_2_* resource family was constructed by crossing homozygous blue-shelled Dongxiang (O*LC/O*LC) males, which has been verified by a test cross, and brown-shelled Dongxiang (O*N/O*N) females. All *F_1_* hens laid blue shelled eggs and individual egg color phenotypes were recorded for all 146 *F_2_* hens.

Six heterozygous (O*LC/O*N) hens were produced by mating one homozygous Dongxiang blue-shelled (O*LC/O*LC) male and one White Leghorn (O*N/O*N) female, and the progeny were used for pyrosequencing analysis.

All animal research was approved by Beijing Administration Committee of Laboratory Animals under the leadership of the Beijing Association for Science and Technology, the approve ID is SYXK (Beijing) 2007–0023.

DNA was extracted from blood using standard phenol/chloroform method. RNA was extracted from the liver and uterus. All the tissue samples for RNA isolation were collected at 3 to 5 hours before the next expected oviposition.

### Linkage analysis

The *F_2_* resource family was used for linkage analysis. A set of 8 markers covering the region anchored by GWAS and *SOX5*, *ev1* were used in the linkage analysis (Figure 2). Marker L1 and L4–L7 were adopted from previous reports [18], [20] and L2, L3 and L8 were mined from the chicken genome assemble (Build 2.1) at http://genome.ucsc.edu/cgi-bin/hgGateway. Fifteen SNP markers (L9–L23) between L4 and L5 were added to narrow the mapping region. Primers and genotyping methods for all markers were present in Table S1. CRI-MAP 2.4 was used for linkage analysis [44]. The TWO-POINT option was used to calculate the recombination fractions between loci as well as corresponding LOD-scores. The CHROMPIC option was used to find unlikely double recombinants.

### Expression analysis

Total RNA was extracted from the uterus using Trizol reagent (TianGen, Dalian, China), followed by synthesis of cDNA from 2 µg of RNA using M-MLV reverse transcriptase (Promega, CA, USA). Five pairs of primer were designed for the four candidate genes (*SLCO1C1*, *SLCO1B3*, *LOC418189* and *SLCO1A2*) and housekeeping gene *GAPDH* using Primer 5.0 for RT-PCR (Table S4) and all primer pairs were designed to span an intron at least. Expression analysis of the four candidate genes was performed in the uterus of blue-shelled (n = 16) and brown-shelled (n = 16) Dongxiang chickens. RT-PCR amplification conditions were as follows: 94°C for 5 min, followed by 36 cycles of amplification (94°C for 30 s, 58°C for 30 s, 72°C for 20 s) and one cycle of 72°C for 5 min.

Expression of *SLCO1B3* in the uterus was subsequently detected in a set of samples including homozygous blue-shelled (O*LC/O*LC, n = 4), heterozygous blue-shelled (O*LC/O*N, n = 4), and brown-shelled (O*N/O*N, n = 4) Dongxiang chickens, homozygous blue-shelled (O*LC/O*LC, n = 3), heterozygous blue-shelled (O*LC/O*N, n = 3), and brown-shelled (O*N/O*N, n = 6) Lushi chickens, and 4 non-blue-shelled breeds including White Leghorn O*N/O*N, n = 6), Beijing You (O*N/O*N, n = 6), Silkies (O*N/O*N, n = 6), Tibetan chicken (O*N/O*N, n = 6) by real-time PCR with a Bio-Rad CFX96 instrument (Bio-Rad, CA, USA). Samples were run in triplicates using RealMasterMix (SYBR Green I) (Tiangen, Dalian, China). PCR amplifications were carried out in a 20 µL reaction volume containing 1.2 pmol of each primer, 9 µL of 2.5×working concentration RealMasterMix and 1 µL of cDNA in following cycling conditions: 95°C for 2 min, followed by 40 cycles of 95°C for 10 s, 58°C for 10 s, 68°C for 10 s. *GAPDH* is used as endogenous reference gene to normalizing amounts of input cDNA, heterozygous blue-shelled Dongxiang (O*LC/O*N) group was designed as a calibrator. Fold change of every group related to the calibrator was calculated as described in Livak *et al.* [45].

### Pyrosequencing

Tissues (the uterus and liver) were collected from the six blue-eggshell heterozygotes. Total RNA was extracted from the uterus and liver with trizol (Tiangen, Dalian, China). The RNA quality was controlled using NanoVue plus spectrophotometer (GE Healthcare, USA). The first-strand cDNA synthesis used M-MLV (Promega, CA, USA) with the 18 hexamers. A fragment containing the SNP (*g. 67334934* G>T) in exon 5 was amplified with forward (CATGTTGCGAGGAATTGGTG) and reverse (TTCCTTAGCAAAATCGTCAAGATA) primers. The relative expression of the two allele (O*LC or O*N) transcripts in heterzygotes was scored by analyzing the SNP (*g. 67334934* G>T) by pyrosequencing. A pyro-seq primer (CGTCAAGATAAGAGATGCC) was used as the sequencing primer and all steps were performed according to manufacturer's protocol. All samples were analyzed in triplicates.

### Fluorescent *in situ* hybridization

The uterus from a 60-week-old egg laying blue-shelled hen and a non-blue-shelled hen were collected and fixed in 4% paraformaldehyde in phosphate buffered saline (PBS) for 24 hours at room temperature. Fixed uterus was embedded in irrigation solution PBS for six hours to eliminate 4% paraformaldehyde. Then slides were dehydrated in increasing concentrations of ethanol (50%, 70%, 80%, 90%, 95% for 1.5 hours each and 100% for 2 hours) followed by transparentizing in two clearing agents respectively of xylene for 15 minutes each. After transparentizing, the slides were pretreated by the mixture of xylene and low melting paraffin for 30 minutes then were directly transferred into pure melting paraffin (58°C) twice for 3 hours each.

The cDNA probe 5′-AACTCTGGCTGAACGCATCT-3′ were labeled by 6-FAM and were synthesized from mRNA of *SLCO1B3* (XM416418.2) by Boxing Bio-engineering Limited Company (Boxing, Guangdong, China). The *in situ* hybridization was then carried out according to the instruction of the FISH Detection Kit (Boxing, Guangdong, China). Imaging was performed using a fluorescence microscope equipped with vision software.

### Resequencing of *SLCO1B3*

Twenty-four kilobases fragment (GenBank accession No. JN020139) covering the whole *SLCO1B3* was resequenced using a panel of ten birds from 5 blue-shelled (O*LC/O*LC) and 5 brown-shelled (O*N/O*N) Dongxiang chickens. Seventeen primer pairs used to generate overlapping PCR amplicons ranging from approximately 800 bp to 2000 bp in size were listed in Table S5. The PCR amplifications were performed in a total volume of 50 µL containing 5 µL of 10×Taq polymerase buffer, 10 mmol of each deoxynucleotide triphosphate (dNTP), 20 pmol of each primer, 2.5 U *Taq* DNA polymerase (HT-biotech, Beijing, China), and 50 ng genomic DNA. All purified PCR products were directly sequenced in both directions using the same primers. The sequences were assembled and analyzed for polymorphisms using the ChromasPro 1.5 or BLAST program in UCSC (http://genome.ucsc.edu/cgi-bin/hgBlat?command=start).

### Rapid amplification of cDNA end (RACE) of *SLCO1B3*

In order to analyze the 5′ and 3′ untranslated regions (UTR) of the *SLCO1B3* gene, RACE experiments were performed on 2 µg total RNA extracted from the uterus of a homozygous blue-shelled (O*LC/O*LC) and a brown-shelled (O*N/O*N) Dongxiang chicken using 5′ and 3′-Full RACE Kit (Takara, Dalian, China), according to the manufacturer's instructions. 5′ and 3′ UTR of *SLCO1B3* gene transcripts were amplified by nested PCR with gene specific (Table S4) and adaptor primers (Table S4) for the first and second amplifications of 5′ and 3′ UTR respectively. First and second PCR amplifications were carried out in a 50 µL reaction volume containing 20 pmol of each primer, 5 µL of 10× LA PCR buffer (Mg^2+^ plus), 2.5 U of LA Taq (Takara, Dalian, China), 20 mM of each dNTP and 1–2 µL of cDNA or 1st PCR product. RACE products were cloned to pMD-18 vector (Takara, Dalian, China), and then sequenced in both directions.

### Long-range PCR

A long-range PCR amplification with 1B3_5F & 5R primer pair (Table S5) was performed in volumes of 50 µL containing 5 µL of 10× LA PCR buffer (Mg^2+^ plus), 2.5 U of LA Taq (Takara, Dalian, China), 20 mM of each dNTP, 20 pmol of each primer and 50 ng genomic DNA. The PCR condition was as follow: 94°C for 3 min followed by 33 cycles of 94°C for 30 s, 58°C for 30 s, 72°C for 5 min, and a final extension at 72°C for 10 min. The PCR product was completely sequenced using the other three pairs of bridging primers (Table S5) besides the 1B3_5F & 5R.

### MassARRAY analysis

Twenty-one SNPs found in resequencing of *SLCO1B3* were used to analyze the genetic variants of *SLCO1B3* (Table S6). SNP markers were genotyped by iPLEX SEQUENOM MassARRAY platform (Sequenom, CA, USA). This genotyping system used single-base extension reactions to create allele-specific products that are separated automatically and scored in a matrix-assisted laser desorption ionization/time of flight mass spectrometer. Primer design was performed using MassARRAY Assay Design software (v3.1) according to Sequenom's instructions. Multiplex PCR amplification of amplicons containing SNPs of interest was performed using HotStart Taq Polymerase (Qiagen, CA, USA) with 12 ng genomic DNA. Assay data were analyzed using Sequenom TYPER software (v3.4).

### Diagnostic genotyping test of *EAV-HP* insertion

The retrovirus insertion was genotyped with a mix of three primers: the primer “test-nor-up” 5′- TTTGACCAGCGTAGATAA-3′ and “test-nor-down” 5′-ATGTTAGCAGTGTAGTTG-3′ were located in the wild type genomic sequence of *SLCO1B3*, the primer “test-eav” 5′-TAGGTTCCGAACGCGATGT-3′ was located in the *gag* region of the inserted retroviral sequence (Figure S3). The PCR amplifications were preformed in a total volume of 25 µL containing 2.5 µL of 10×Taq polymerase buffer, 5 mmol of each deoxynucleotide triphosphate (dNTP), 10 pmol of each primer, 1.25 U Taq DNA polymerase (HT-biotech, Beijing, China), and 50 ng genomic DNA in the following condition: 94°C for 5 min, followed by 36 cycles of 94°C for 30 s, 58°C for 30 s, 72°C for 20 s, and a final extension at 72°C for 5 min. The PCR products was separated by 2% agarose gel electrophoresis, and the length of target fragment was 340 bp for test-nor-up and test-nor-down, and 425 bp for test-nor-up and test-eav, respectively (Figure S3).

### *EAV-HP* insertion site sequencing

Two pairs of PCR primers (EAVIS-1F, EAVIS-1R, EAVIS-2F and EAVIS-2R, Table S5) were designed for amplifying 5′ and 3′ end of *EAV-HP* junction regions. The PCR condition was as follow: 94°C for 3 min followed by 33 cycles of 94°C for 30 s, 57°C for 30 s and 54°C for 40 s, respectively, 72°C for 45 s, and a final extension at 72°C for 10 min. The PCR products were sequenced bidirectionally using the PCR primers.

## Supporting Information

Figure S1 The sequencing results of 5′RACE for *SLCO1B3* in blue-shelled Dongxiang chicken. Sequences showed in blue color are newly obtained 5′ UTR of *SLCO1B3* which has been submitted to GenBank with accession No. JN381032. The underlined sequences are transcription from *EAV-HP* insertion.
(TIF)

Figure S2 Expression analysis for *SLCO1B3* in white-shelled and blue-shelled ducks by RT-PCR.
(TIF)

Figure S3 Diagnostic genotyping test of *EAV-HP* insertion. (A) primers information for diagnostic genotyping test of *EAV-HP* insertion. (B) results for diagnostic genotyping test of *EAV-HP* insertion in blue-shelled and non-blue-shelled Dongxiang chickens. Single 425 bp band represents homozygous blue-shelled chickens, single 340 bp band corresponds to non-blue-shelled chickens, and two amplifications are heterozygous chickens.
(TIF)

Table S1 Information of markers used in linkage analysis.
(DOCX)

Table S2 Two point linkage analysis with markers in L4–L5 interval.
(DOCX)

Table S3 Haplotype frequency analysis of all the breeds or populations.
(DOCX)

Table S4 Primers used in expression analysis, 5′ and 3′ RACE of candidate genes.
(DOCX)

Table S5 Primer sequences used in resequencing of *SLCO1B3* and *EAV-HP*.
(DOCX)

Table S6 Information of SNPs in *SLCO1B3* gene.
(DOCX)
