# Supplementary figures and images for: An EAV-HP Insertion in 5′ Flanking Region of SLCO1B3 Causes Blue Eggshell in the Chicken
Source: PLoS Genet. 2013 Jan 24;9(1):e1003183. doi: 10.1371/journal.pgen.1003183 (PMC3554524; doi:10.1371/journal.pgen.1003183)

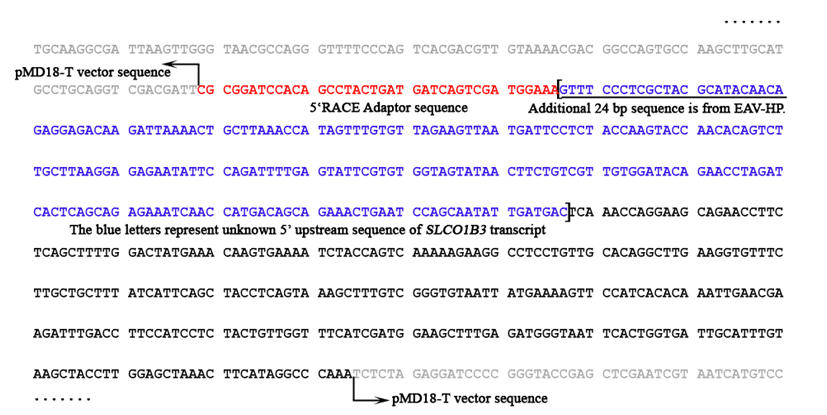

Supplement: Figure S1 — The sequencing results of 5′RACE for SLCO1B3 in blue-shelled Dongxiang chicken. Sequences showed in blue color are newly obtained 5′ UTR of SLCO1B3 which has been submitted to GenBank with accession No. JN381032. The underlined sequences are transcription from EAV-HP insertion. (TIF) [file pgen.1003183.s001.tif]

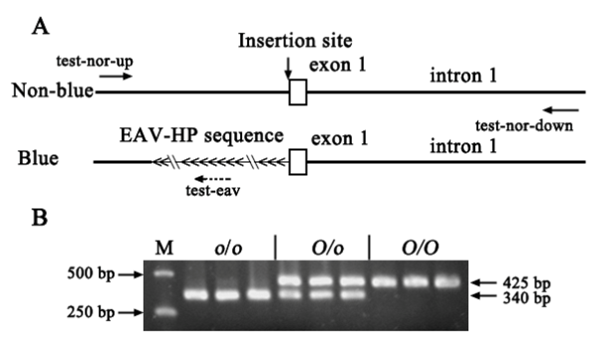

Supplement: Figure S3 — Diagnostic genotyping test of EAV-HP insertion. (A) primers information for diagnostic genotyping test of EAV-HP insertion. (B) results for diagnostic genotyping test of EAV-HP insertion in blue-shelled and non-blue-shelled Dongxiang chickens. Single 425 bp band represents homozygous blue-shelled chickens, single 340 bp band corresponds to non-blue-shelled chickens, and two amplifications are heterozygous chickens. (TIF) [file pgen.1003183.s003.tif]
