# Supplementary material for: An EAV-HP Insertion in 5′ Flanking Region of SLCO1B3 Causes Blue Eggshell in the Chicken
Source: PLoS Genet. 2013 Jan 24;9(1):e1003183. doi: 10.1371/journal.pgen.1003183 (PMC3554524; doi:10.1371/journal.pgen.1003183)
Supplement: Table S1 — Information of markers used in linkage analysis. (DOCX) [file pgen.1003183.s004.docx]

**Table S1**. Information of markers used in linkage analysis

| name | description^a^ | accession No. ^b^ | primer sequence (5’ to 3’) | genotyping method |
| --- | --- | --- | --- | --- |
| L1 | *g.65699879TG*  (12_15) | HM461742 | F: TGAGATTTCCTCGCTTTT  R: CTTGCTGTCCACTGTTTA | 12% PAGE gel electrophoresis |
| L2 | *g.66227251G>T* | rs15295129 | F: ACAGTGATTGGCAAAGGT  R: GGTAGTTGAGAAGGGTAAA | PCR-RFLP, HaeⅢ |
| L3 | *g.66754390A>T* | rs15296240 | F: GCAGAGTGGTGGTGAAGT  R: AACAGAACAGATGGCTGA | PCR-RFLP, PshBⅠ |
| L4 | *g.67296991T>A* | rs15297163 | F: TCATTTCCAAAGCCCAATC  R: CATCAATAACGCCAACCA | PCR-RFLP, *BsmI* |
| L5 | *g.67419892_67419904del13* | ss244244378 | F: ATCTATAAAGGAGCAAGG  R: ATGAGGGTAAGAGGACAC | PCR-SSCP |
| L6 | *g.68170663T>G* | ss244255171 | F: GGTGTTTCTGTCGGGTAT  R:TTTCAGGCTGCTTCATTT | PCR-RFLP, *DraI* |
| L7 | *g.68680338TCTG*  (7_13) | HM461745 | F: TTTCTGTATTGCCTTGCA  R: CATTTGTTCCGTTCCACT | 12% PAGE gel electrophoresis |
| L8 | *g.69047664GGAA*(11-18) | HM461746 | F: AGCGGTCTTTGTTTCCAG  R: TCATCAGGTCCAACTTCC | 12% PAGE gel electrophoresis |
| L9 | *g.67308596G>A* | rs15297217 | - | MALDI-TOF MS |
| L10 | *g.67318333G>C* | rs15297241 | - | MALDI-TOF MS |
| L11 | *g.67320217A>G* | ss469105378 | - | MALDI-TOF MS |
| L12 | *g.67320779A>G* | ss469105379 | - | MALDI-TOF MS |
| L13 | *g.67336599C>T* | ss469105391 | - | MALDI-TOF MS |
| L14 | *g.67336867A>T* | ss469105392 | - | MALDI-TOF MS |
| L15 | *g.67337145A>G* | ss469105393 | - | MALDI-TOF MS |
| L16 | *g.67338442C>G* | ss469105394 | - | MALDI-TOF MS |
| L17 | *g.67339848A>G* | ss469105396 | - | MALDI-TOF MS |
| L18 | *g.67340370C>T* | ss469105397 | - | MALDI-TOF MS |
| L19 | *g.67342640A>G* | rs14837781 | - | MALDI-TOF MS |
| L20 | *g.67359366A>G* | rs14837812 | - | MALDI-TOF MS |
| L21 | *g.67363727A>C* | rs15297326 | - | MALDI-TOF MS |
| L22 | *g.67396943C>T* | rs15297441 | - | MALDI-TOF MS |
| L23 | *g.67416784A>T* | rs13879411 | - | MALDI-TOF MS |

^a^ Nucleotide numbering according to May 2006 chicken (Gallus gallus) v2.1 draft assembly (<http://genome.ucsc.edu>).

^b^ The markers data reported in this paper have been submitted to the NCBI GenBank and dbSNP database (<http://www.ncbi.nlm.nih.gov/genbank/> and <http://www.ncbi.nlm.nih.gov/snp/>).
