# Supplementary material for: An EAV-HP Insertion in 5′ Flanking Region of SLCO1B3 Causes Blue Eggshell in the Chicken
Source: PLoS Genet. 2013 Jan 24;9(1):e1003183. doi: 10.1371/journal.pgen.1003183 (PMC3554524; doi:10.1371/journal.pgen.1003183)
Supplement: Table S2 — Two point linkage analysis with markers in L4–L5 interval. (DOCX) [file pgen.1003183.s005.docx]

**Table S2.** Two point linkage analysis with markers in L4-L5 interval

| Marker | Recombination rate | LOD | Position | gene^a^ |
| --- | --- | --- | --- | --- |
| L_9_ | 0 | 11.74 | 67308696 | *SLCO1C1* |
| L_10_ | 0 | 12.04 | 67318333 | - |
| L_11_ | 0 | 18.06 | 67320217 | *SLCO1B3* |
| L_12_ | 0 | 18.06 | 67320779 |  |
| *EAV-HP* | 0 | 18.06 | 67324641-67324642 |  |
| L_13_ | 0 | 18.06 | 67336599 |  |
| L_14_ | 0 | 18.06 | 67336867 |  |
| L_15_ | 0 | 18.06 | 67337145 |  |
| L_16_ | 0 | 18.06 | 67338442 |  |
| L_17_ | 0 | 18.06 | 67339848 |  |
| L_18_ | 0 | 18.06 | 67340370 |  |
| L_19_ | 0 | 18.06 | 67342640 |  |
| L_20_ | 0 | 5.12 | 67359366 | *LOC418189* |
| L_21_ | 0 | 18.06 | 67363727 |  |
| L_22_ | 0 | 6.02 | 67396943 | *SLCO1A2* |
| L_23_ | 0.02 | 10.00 | 67416784 | - |

^a^ L9 locates in *SLCO1C1* gene. L10 and L23 are inter gene markers. L11-L19 locate in *SLCO1B3* gene. L20 and L21 are in *LOC418189* gene*.* L22 locates in *SLCO1A2* gene.
