# Supplementary material for: An EAV-HP Insertion in 5′ Flanking Region of SLCO1B3 Causes Blue Eggshell in the Chicken
Source: PLoS Genet. 2013 Jan 24;9(1):e1003183. doi: 10.1371/journal.pgen.1003183 (PMC3554524; doi:10.1371/journal.pgen.1003183)
Supplement: Table S3 — Haplotype frequency analysis of all the breeds or populations. (DOCX) [file pgen.1003183.s006.docx]

**Table S3.** Haplotype frequency analysis of all the breeds or populations^a^

| Haplo ID | Haplotype^b^ | All | Dongxiang blue shelled | Lushi  blue shelled | Araucana blue shelled | Dongxiang brown shelled | Lushi brown shelled | Dwarf | Luxi Game | White Leghorn | Silkies | Tibetan | Red jungle fowl | Rhode Island Red |
| --- | --- | --- | --- | --- | --- | --- | --- | --- | --- | --- | --- | --- | --- | --- |
| Haplotype1 | GGT-GACCACGTCGCAACCACG | 0.169 |  |  |  | 0.630 |  | 0.063 |  |  | 0.233 | 0.268 |  | 0.157 |
| Haplotype2 | AAT-CATCGCGCCGCAACCACG | 0.157 |  |  |  |  | 0.167 | 0.467 | 0.550 |  |  | 0.161 | 0.217 |  |
| Haplotype3 | GGT-GATCACGCCGCAACCACG | 0.103 |  |  |  |  | 0.150 | 0.050 | 0.067 | 0.350 | 0.050 | 0.231 |  |  |
| **Haplotype4** | **AAC+GGCTATTTTTTTGGTGTA** | **0.080** | **0.924** | **0.828** |  |  |  |  |  |  |  |  |  |  |
| Haplotype5 | GGT-CACCACGCCGCAACTACG | 0.056 | 0.030 | 0.138 |  | 0.220 | 0.100 |  |  |  |  |  |  |  |
| Haplotype6 | GGT-GATCACGTCGCAACCACG | 0.051 |  |  |  |  |  | 0.304 |  |  |  |  |  | 0.410 |
| Haplotype7 | GGC-CATCACGTCGCAACCACG | 0.045 |  |  | 0.200 |  |  | 0.063 |  | 0.100 |  | 0.054 |  | 0.200 |
| Haplotype8 | GGC-CATCGCGCCGCAGGTACA | 0.037 |  |  |  |  |  |  |  | 0.383 |  |  |  |  |
| Haplotype9 | GGT-GGTCGCGTCGCAACTACG | 0.024 |  |  |  |  |  |  |  |  | 0.167 |  |  |  |
| Haplotype10 | GGT-GGTCGCGCCGCAACCACG | 0.023 |  |  |  |  |  |  |  |  | 0.283 |  |  |  |
| Haplotype11 | GGT-CACCACGCCGCAACCACG | 0.022 |  |  |  | 0.150 |  |  |  |  |  |  |  |  |
| Haplotype12 | GGT-GGTCGCGCCGCAGGTACA | 0.014 |  |  |  |  |  |  |  | 0.133 |  |  |  |  |
| Haplotype13 | GGT-GATCGCGTCGCAACCACG | 0.014 |  |  |  |  | 0.217 |  |  |  |  |  |  |  |
| **Haplotype14** | **GGT+GATCACGCCGCAACCACG** | **0.011** |  |  | **0.700** |  |  |  |  |  |  |  |  |  |
| Haplotype15 | GGT-GGCCACGTCGCAACCACG | 0.000 | 0.046 |  |  |  |  |  |  |  |  |  |  |  |
| Haplotype16 | AAT-CATCACGCCGCAACCACG | 0.000 |  |  | 0.100 |  |  |  |  |  |  |  |  |  |
| Haplotype17 | AAC-GACCGCGTCGCAACCGCG | 0.000 |  |  |  |  | 0.083 |  |  |  |  |  |  |  |
| Haplotype18 | GGT-GACCGCTTCGCAACCACG | 0.000 |  |  |  |  |  |  | 0.117 |  |  |  |  |  |
| Haplotype19 | GGT-GGCCGCTTCGCAACTACG | 0.000 |  |  |  |  |  |  | 0.100 |  |  |  |  |  |
| Haplotype20 | AAT-CATCGCTTTTTTACCACA | 0.000 |  |  |  |  |  |  |  |  | 0.133 |  |  |  |
| Haplotype21 | GGT-GACCACGTCGCAAGTGTA | 0.000 |  |  |  |  |  |  |  |  | 0.100 |  |  |  |
| Haplotype22 | AAT-GGTCACGTCGCAACCACG | 0.000 |  |  |  |  |  |  |  |  |  | 0.089 |  |  |
| Haplotype23 | GAC-GACCACGTCGCAACCACG | 0.000 |  |  |  |  |  |  |  |  |  | 0.054 |  |  |
| Haplotype24 | GGT-GACCGCGTCGCAACCACG | 0.000 |  |  |  |  |  |  |  |  |  |  | 0.104 |  |
| Haplotype25 | GGT-GATCGCGCCGCAACCACG | 0.000 |  |  |  |  |  |  |  |  |  |  | 0.067 |  |
| Haplotype26 | GGT-GACCGCGTCGCTACCACG | 0.000 |  |  |  |  |  |  |  |  |  |  | 0.063 |  |
| Haplotype27 | GGT-GACCACGTCTTTGGTGTG | 0.000 |  |  |  |  |  |  |  |  |  |  | 0.050 |  |
| Haplotype28 | GGT-GATCACGCCGCAACCATG | 0.000 |  |  |  |  |  |  |  |  |  |  |  | 0.167 |

^a^ Haplotypes with frequency>0.05 in each breed or populations are mostly displayed in the table.

^b^ Haplotypes were constructed by PHASE v2.0 [Ref. S1].

Ref. S1. Stephens M, Donnelly P (2003) A comparison of Bayesian methods for haplotype reconstruction from population genotype data. Am J Hum Genet 73: 1162–1169.
