# Supplementary material for: An EAV-HP Insertion in 5′ Flanking Region of SLCO1B3 Causes Blue Eggshell in the Chicken
Source: PLoS Genet. 2013 Jan 24;9(1):e1003183. doi: 10.1371/journal.pgen.1003183 (PMC3554524; doi:10.1371/journal.pgen.1003183)
Supplement: Table S4 — Primers used in expression analysis, 5′ and 3′ RACE of candidate genes. (DOCX) [file pgen.1003183.s007.docx]

**Table S4.** Primers used in expression analysis, 5’ & 3’ RACE of candidate genes

| experiment | primer ID | primer (5’-3’) | fragment length (bp) |
| --- | --- | --- | --- |
| RT-PCR | 1C1_F | TAGATACTACTTGCCTGAAATG | 157 |
|  | 1C1_R | AGAACCCACAGGACAGCA |  |
|  | 1B3_F | TTTTGGGGCACTGATTGA | 205 |
|  | 1B3_R | GGGAGTCCACCTAGTATT |  |
|  | 189_F | CAAAGAGAGAGCTGTACC | 182 |
|  | 189_R | GGGAGTCCACCTAGTATT |  |
|  | 1A2_F | GTCTGGCTGGGTGTAAGG | 188 |
|  | 1A2_R | AATGAAGCTGCCGACTAA |  |
|  | GAPDH_F | TATCTTCCAGGAGCGTGA | 130 |
|  | GAPDH_R | CTCATTGTCATACCAGGA |  |
| Real-time quantitative PCR in chicken | 1B3_F | TTTTGGGGCACTGATTGA | 205 |
|  | 1B3_R | GGGAGTCCACCTAGTATT |  |
|  | GAPDH_F | TATCTTCCAGGAGCGTGA | 130 |
|  | GAPDH_R | CTCATTGTCATACCAGGA |  |
| *SLCO1B3* 5’RACE | 5’ GSP1 | GATGTGGAGAGCAGGGATTTATG |  |
|  | 5’ GSP2 | TGGGCCTATGAAGTTTAGCTCCA |  |
|  | 5’Outer | CATGGCTACATGCTGACAGCCTA |  |
|  | 5’Inner | CGCGGATCCACAGCCTACTGATGATCAGTCGATG |  |
| *SLCO1B3* 3’RACE | 3’GSP | TTTTGGGGCACTGATTGA |  |
|  | 3’Outer | TACCGTCGTTCCACTAGTGATTT |  |
|  | 3’Inner | CGCGGATCCTCCACTAGTGATTTCACTATAGG |  |
| RT-PCR for *SLCO1B3* in duck | Duck-1F | TTTTGGTGCACTGATTGA | 205 |
|  | Duck-1R | AGGTCTGGAGTTCTTATT |  |
|  | DGAPDH_F | ATACACAGAGGACCAGGTTG | 130 |
|  | DGAPDH_R | ATACTCGTTGTCATACCAGG |  |
