# Supplementary material for: An EAV-HP Insertion in 5′ Flanking Region of SLCO1B3 Causes Blue Eggshell in the Chicken
Source: PLoS Genet. 2013 Jan 24;9(1):e1003183. doi: 10.1371/journal.pgen.1003183 (PMC3554524; doi:10.1371/journal.pgen.1003183)
Supplement: Table S5 — Primer sequences used in resequencing of SLCO1B3 and EAV-HP. (DOCX) [file pgen.1003183.s008.docx]

**Table S5.** Primer sequences used in resequencing of *SLCO1B3* and *EAV-HP*

| experiment | primer ID | primer sequence (5’-3’) | fragment size (bp) | anneal temperature（℃） |
| --- | --- | --- | --- | --- |
| resequencing *SLCO1B3* | 1B3_1F | GAGTTACATGCTCTATCCCTT | 953 | 57 |
|  | 1B3_1R | GACTCTGTCATTATGCTCCAA |  |  |
|  | 1B3_2F | AATTGGCACGGGTGCTAGAGT | 792 | 57 |
|  | 1B3_2R | CACAGAACGGAGACCAGGAGT |  |  |
|  | 1B3_3F | AGCAGGACAATGGCAGTATGT | 1053 | 57 |
|  | 1B3_3R | TCAAGATTTGGATGGAGGTTC |  |  |
|  | 1B3_4F | AAAGCATCCCAAACTTCAATG | 1037 | 57 |
|  | 1B3_4R | AGGAAATGGTAGCCACCTCAA |  |  |
|  | 1B3_5F | TGCCTTGTAACTTTGAGGTGG | 1809^*a^ | 57 |
|  | 1B3_5R | GGTTTCTTCGGTAATTTAGGGTAT |  |  |
|  | 1B3_6F | TTTGACCAGCGTAGATAA | 1708^*a^ | 57 |
|  | 1B3_6R | ATGCCACAAGAAGATAAGA |  |  |
|  | 1B3_7F | AAGGCTTCACACCTATCC | 1744 | 53.8 |
|  | 1B3_7R | CTTCTTGCAACACTACAC |  |  |
|  | 1B3_8F | TGTTGCTGGGAATAGTCG | 1158 | 60 |
|  | 1B3_8R | CCCAAACACATTGGTATC |  |  |
|  | 1B3_9F | TACTCACAGTTAGGTCTT | 1941 | 53 |
|  | 1B3_9R | CATGGTCTATGCCTGTTA |  |  |
|  | 1B3_10F | ATCTACATTTCCGCAACA | 2069 | 55 |
|  | 1B3_10R | GAGGGAGACTGAGACTGG |  |  |
|  | 1B3_11F | GAGGGTGGGCTCTGTATC | 1684 | 57 |
|  | 1B3_11R | GGAAATGCGAGTATTGTAG |  |  |
|  | 1B3_12F | GTTATAAGTATGAGACAG | 1787 | 49 |
|  | 1B3_12R | AGCAGTAACAATGAACAC |  |  |
|  | 1B3_13F | AGAATGCTCAGAGACTAA | 1569 | 57 |
|  | 1B3_13R | CATGTACTTCGGCTTGTA |  |  |
|  | 1B3_14F | AAGCCAACTCATTTATCTC | 1774 | 59.5 |
|  | 1B3_14R | TTCGCATTTCATCTCACT |  |  |
|  | 1B3_15F | TGAGATGAAATGCGAAGC | 1700 | 55.9 |
|  | 1B3_15R | AAGCAACCCACTAGAACC |  |  |
|  | 1B3_16F | CATTCTCCTTACATTTGC | 1859 | 59.5 |
|  | 1B3_16R | CTTCCCTCTCATTGGTAA |  |  |
|  | 1B3_17F | TCTTGGCTCTTCACTCAG | 1471 | 62 |
|  | 1B3_17R | ACTTGTCCCAGAACGATA |  |  |
| sequencing *EAV-HP* | EAV_1F | ACATGTATTTTGGGACCTTCAAC | 4314 | 58 |
|  | EAV_1R | TGGTACTTGGTAGAGGAATCATT |  |  |
|  | EAV_2F | GTAGCTCATCACCGCAGTCAG | 2349 | 58 |
|  | EAV_2R | ACAACGAAGAGTCGCAGAGGC |  |  |
|  | EAV_3R | AGGGAGGGTTGTATTTAGAGC | 629 | 58 |
|  | EAV_3R | GACAGTTAGGACCTTGCTTTA |  |  |
| sequencing *EAV-HP* insertion site | EAVIS-1F, | TAGTTGAACACCCACTTTT | 479^b^ | 57 |
|  | EAVIS-1R | TTGCCTCACTTACTCCTG |  |  |
|  | EAV IS-2F | CGCCACCTCGCAGGCTCTTA | 599^b^ | 54 |
|  | EAVIS-2R | ATACTACCACACGAATACTC |  |  |

^a^ The fragments containing the insertion site of *EAV-HP*.

^b^ PCR product to determine *EAV-HP* insertion site.
