# Supplementary material for: An EAV-HP Insertion in 5′ Flanking Region of SLCO1B3 Causes Blue Eggshell in the Chicken
Source: PLoS Genet. 2013 Jan 24;9(1):e1003183. doi: 10.1371/journal.pgen.1003183 (PMC3554524; doi:10.1371/journal.pgen.1003183)
Supplement: Table S6 — Information of SNPs in SLCO1B3 gene. (DOCX) [file pgen.1003183.s009.docx]

**Table S6.** Information of SNPs in *SLCO1B3* gene

| Name | Description^a^ |
| --- | --- |
| 1B3_1 | *g.67320217A>G* |
| 1B3_2 | *g.67320779A>G* |
| 1B3_3 | *g.67322980C>T* |
| 1B3_4 | *g.67325964G>C* |
| 1B3_5 | *g.67328567A>G* |
| 1B3_6 | *g.67330251C>T* |
| 1B3_7 | *g.67330635C>T* |
| 1B3_8 | *g.67332494A>G* |
| 1B3_9 | *g.67333488C>T* |
| 1B3_10 | *g.67334934G>T* |
| 1B3_11 | *g.67335932T>C* |
| 1B3_12 | *g.67336084C>T* |
| 1B3_13 | *g.67336453G>T* |
| 1B3_14 | *g.67336599C>T* |
| 1B3_15 | *g.67336867A>T* |
| 1B3_16 | *g.67337145A>G* |
| 1B3_17 | *g.67338442C>G* |
| 1B3_18 | *g.67339003C>T* |
| 1B3_19 | *g.67339848A>G* |
| 1B3_20 | *g.67340370C>T* |
| 1B3_21 | *g.67342640A>G* |

^a^ Nucleotide numbering according to May 2006 chicken (Gallus gallus) assembly (<http://genome.ucsc.edu>).
